# Effects of Dietary Fiber Supplementation on Chronic Constipation in the Elderly: A Systematic Review and Meta-Analysis of Randomized Controlled Trials

**DOI:** 10.3390/foods14132315

**Published:** 2025-06-30

**Authors:** Na Xi, Xiao Yang, Jie Liu, Hao Yue, Ziyuan Wang

**Affiliations:** 1 Key Laboratory of Geriatric Nutrition and Health (Beijing Technology and Business University), Ministry of Education, Beijing 100048, China; 2 National Center of Technology Innovation for Grain Industry (Comprehensive Utilization of Edible By-Products), Beijing Technology and Business University, Beijing 100048, China; 3 Institute of Food & Nutrition Science and Technology, Shandong Academy of Agricultural Sciences, Jinan 250100, China

**Keywords:** dietary fiber, elderly, chronic constipation, gut microbiota, meta-analysis

## Abstract

Chronic constipation is common among older adults, significantly reducing quality of life and increasing healthcare burden. While dietary fiber is a traditional intervention for constipation, its efficacy in elderly populations remains inconclusive. The purpose of this study was to investigate the effects of dietary fiber on stool frequency, frequency of laxative or enema use, intestinal bifidobacteria concentration, stool dry weight, and adverse events in elderly patients with chronic constipation through a systematic review and meta-analysis of randomized controlled trials (RCTs). A comprehensive search of Embase, Web of Science, PubMed, Scopus, and The Cochrane Library databases was performed to identify eligible studies. Seven RCTs involving 187 participants were included. The meta-analysis found no significant improvement in stool frequency with dietary fiber supplementation (SMD = 0.25, 95% CI [−0.488, 0.988], *p* = 0.507). However, dietary fiber significantly reduced the frequency of laxative or enema use (SMD = −1.224, 95% CI [−1.786, −0.662], *p* = 0.000) and increased fecal bifidobacteria concentrations (SMD = 5.142, 95% CI [3.716, 6.568], *p* = 0.000). These findings suggest that dietary fiber supplementation may reduce reliance on laxatives and enhance intestinal microbiota in elderly individuals with chronic constipation. Further robust and clear randomized controlled trials are needed to more accurately determine the most effective dosage and duration of use.

## 1. Introduction

Chronic constipation is a routine, multifactorial, and heterogeneous gastrointestinal disease [1]. It is typically defined as infrequent bowel movements accompanied by excessive straining, a sensation of incomplete evacuation, failed or prolonged attempts to defecate, abdominal distension, and hard stool consistency [2,3,4]. The global prevalence of chronic constipation is estimated at approximately 20%, with a higher incidence observed among the elderly [5,6]. A population-based study has shown that the incidence of chronic constipation tends to increase with age, particularly after age 65 [7]. Moreover, constipation is more prevalent among elderly women than men [8,9]. A study assessing constipation in the elderly found that the prevalence rates further increased to 34% for women and 26% for men aged 85 years and older [10]. While its precise etiology remains unclear in many cases, several contributing factors have been identified, including lifestyle changes, psychosocial conditions, dietary patterns [5], medication use, and comorbidities [11,12]. If chronic constipation is not addressed in a timely manner, it may lead to more severe anorectal complications, such as anal fissures, fecal impaction, rectal bleeding, and hemorrhoids [13,14]. These complications can cause restlessness and anxiety, which will further increase medical costs and negatively impact patients’ health-related quality of life [15]. Constipation is a prevalent and serious health issue among the elderly, which has a considerable adverse effect on their quality of life and healthcare resource utilization [16].

Managing constipation in the elderly can be particularly challenging due to multiple interacting factors. Treatment typically involves a multimodal approach, incorporating dietary modifications, physical activity, and pharmacologic therapies [17,18]. Several drug therapeutic approaches are available for managing chronic constipation, ranging from traditional laxatives to more recently developed pharmaceutical options such as secretagogues, chenodeoxycholate, and bile acid agents [19]. However, despite the wide variety of laxatives available, approximately half of the patients with constipation do not achieve satisfactory results with their use [20]. Additionally, the high cost and adverse side effects of these medications limit their application in the management of constipation [21]. Therefore, dietary adjustments play a significant role in alleviating constipation. Dietary fiber is a key non-pharmacologic intervention in constipation management. It is a polysaccharide in complex carbohydrates that is not easily digested or absorbed by enzymes produced in the body nor can it produce energy [22,23]. Epidemiological studies have consistently indicated that dietary fiber is beneficial for gastrointestinal health [24]. In the colon, dietary fiber is fermented by the microbiota to produce short-chain fatty acids (SCFAs), which accelerate intestinal transit and increase intestinal motility [25,26]. Recent studies have also indicated its potential prebiotic effect, particularly in increasing the abundance of beneficial gut bacteria such as bifidobacteria [27]. Furthermore, high-fiber diets lead to a larger and softer mass in the large intestine. This causes the intestine to contract, moving the contents towards excretion [28]. Some studies have indicated that fiber supplementation results in an increase in stool weight and accelerates intestinal transit, leading to an increase in stool frequency [29,30]. Soluble fiber is effective in increasing defecation frequency, stool weight and consistency, and reducing defecation tenderness and pain, but it does not improve bowel transit [31]. Insoluble fiber, such as bran, shortens intestinal transit time [32]. Notably, dietary fiber found in pulses and whole grains proves highly effective in maintaining regular bowel movements, along with cereal bran, psyllium husk, and methylcellulose preparations [33]. Furthermore, fructans which are soluble fibers, exhibit positive effects on large intestine health, with inulin consumption linked to increased bowel movement frequency [34].

Despite the possible potential benefits of dietary fiber supplementation, evidence on its efficacy in the management of constipation in older adults remains conflicting. Negative effects have been reported: insoluble fiber may worsen symptoms, including abdominal pain and bloating [35]. In addition, although previous meta-analyses have evaluated the impact of fiber on constipation in general adult or mixed-age populations, few have focused solely on elderly people. Therefore, a systematic review and meta-analysis of randomized controlled trials were conducted to evaluate the effects of dietary fiber supplementation on chronic constipation in the elderly. Specifically, this study aimed to assess its impact on stool frequency, frequency of laxative or enema use, intestinal bifidobacteria concentration, stool dry weight, and the occurrence of adverse events among participants. The findings from this study are expected to enhance our understanding of the role of dietary fiber in alleviating chronic constipation in the elderly.

## 2. Materials and Methods

### 2.1. Protocol and Registration

The study adhered to the guidelines outlined in the Preferred Reporting Items for Systematic Reviews and Meta-Analyses (PRISMA) [36,37] (Table S1). The protocol was registered with PROSPERO (CRD42024533190) on 17 April 2024.

### 2.2. Literature Searches

The literature was identified through a search conducted in the Embase, Web of Science, PubMed, Scopus, and The Cochrane Library databases, which covered studies published from 1946 to May 2024. No restrictions were placed on language. The search terms included a combination of MeSH terms and free-text keywords: (constipation OR dyschezia OR colonic Inertia) AND (aged OR elderly) AND (dietary fiber OR dietary fibers OR fibers dietary OR fiber dietary OR wheat bran OR bran wheat OR brans wheat OR wheat brans OR roughage OR roughages) AND (randomized controlled trials). Detailed search strategies for each database are listed in Table S2.

### 2.3. Eligibility Criteria

Studies that met the following criteria were included in this meta-analysis: (1) Study objects: Constipated people of any race, gender, and age over 50 years; Chronic constipation must be diagnosed based on internationally recognized criteria, such as the Rome guidelines, self-reported symptoms, or clinical assessment by a physician. Must have at least one of the following symptoms of constipation: less than three bowel movements per week, dry or granular stools, feeling of incomplete evacuation, need to strain to defecate, need manual assistance to defecate, or have a bowel obstruction. (2) Interventions: Any type of dietary fiber supplement including oral intake, with no restriction on the dose and duration of the intervention. (3) Outcomes: Trials reporting either dichotomous or continuous data on treatment response, stool frequency, stool consistency, stool weight, gastrointestinal symptoms, use of laxatives or enemas, and adverse events. (4) Study design: Only research designs that were randomized controlled trials (RCTs) were included. PICOS criteria for eligibility are presented in Table 1.

Excluded from the analysis were studies that did not meet the following criteria: (1) Any duplicate publications or studies. (2) Studies with incomplete data are excluded, such as conference papers, abstracts, and other sources. (3) The data in the study cannot be converted into the required data format.

### 2.4. Data Extraction

Data on the predefined variables were independently extracted by two investigators and recorded on an Excel sheet. Extracted data included the following items: details of the RCTs’ fundamentals, such as the first author, year, study design, and country of publication; participant characteristics, including sample size, gender, age, and diagnostic criteria for constipation; details of intervention measures, including types of dietary fiber, fiber dosage, form and treatment duration; and outcomes and data related to study design. Discrepancies were resolved by consensus or a third reviewer.

### 2.5. Assessment of Risk of Bias

The quality appraisal of the included trials was conducted using the revised Cochrane risk-of-bias tool for randomized trials (RoB 2.0) [38]. The tool assesses five domains of bias, each comprising signaling questions to determine the risk of bias (Table S3). The articles were independently assessed by a pair of reviewers in accordance with the RoB 2.0. Response options for each query ranged from “yes”, “probably yes” to “no”, “probably no”, and “no information”. Employing the Cochrane RoB 2.0 algorithm, the final RoB rating classified as “high”, “low”, or “some concerns” was determined.

### 2.6. Data Synthesis and Statistical Analysis

Statistical analysis and forest plots were primarily generated using Stata version 17.0. A random-effects model was employed to consolidate the data, aiming to provide a more cautious estimate of the impact of dietary fiber supplementation on chronic constipation, while also accounting for the heterogeneity observed across the trials. Treatment effects, including potential adverse reactions, were evaluated using relative risk (OR) with a 95% confidence interval (CI) for dichotomous outcomes and standardized mean difference (SMD) with a 95% CI for continuous outcomes (such as stool frequency, stool weight, gut microbiota concentrations, and laxative use). Heterogeneity among the studies was assessed utilizing both Cochran’s Q test and the I-squared (*I*^2^), with *I*^2^ values of 25%, 50%, and 75% representing low, moderate, and high heterogeneity levels, respectively. Funnel plot asymmetry and sensitivity analyses were performed using Stata version 17.0. Where feasible, subgroup analyses were conducted based on fiber type, dose, and study size. *p* values less than 0.05 were considered statistically significant.

## 3. Results

### 3.1. Results of the Search

**Figure 1:**
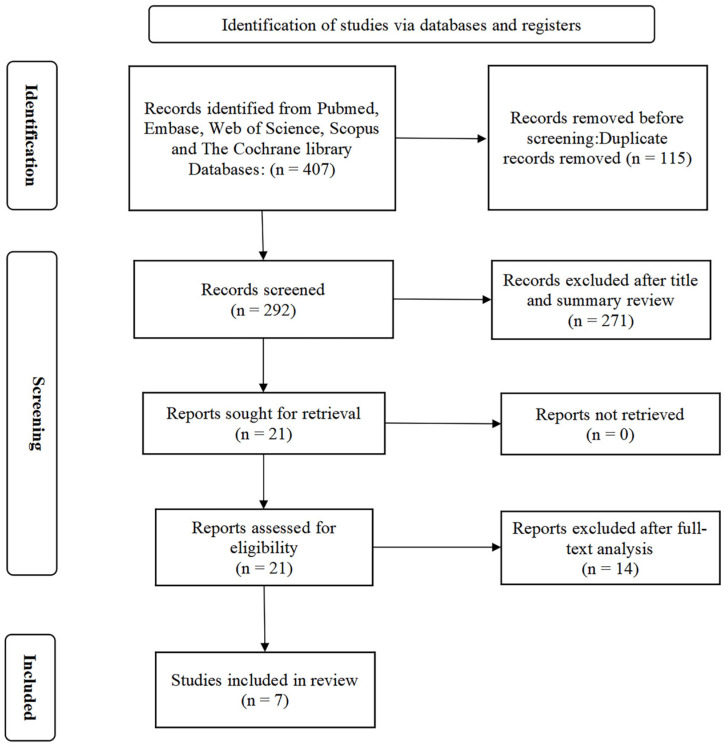
PRISMA flow diagram of the literature search.

A total of 407 articles were retrieved using the search strategy. After removing 115 repetitions using Endnote version X9 software, 292 articles remained. Furthermore, the authors screened the titles and abstracts, and a further 271 studies were excluded in light of the study question and inclusion/exclusion criteria. Following careful screening of the entire texts, only seven studies met the inclusion criteria for the systematic review and meta-analysis [39,40,41,42,43,44,45]. The study selection process is depicted in the PRISMA flow diagram (Figure 1).

### 3.2. Study Characteristics

A total of 187 participants with chronic constipation were included across the seven RCTs, comparing dietary fiber supplementation with placebo/control. All participants were aged over 50 years and were recruited from nursing homes or hospital outpatient settings. Sample sizes ranged from 10 to 60 participants, and intervention durations ranged from 4 to 12 weeks. Three studies were conducted in China, and the remaining four were from Austria, the United States, England, and France. Among these trials, four used a parallel design and three used a crossover design. Dietary fiber supplements were available as liquid, sachets, and dietary mixtures. There was variability in the dose (5–22 g/day) of dietary fiber studied. The effectiveness of six different types of fiber in treating constipation in older people was assessed in the included studies. Fiber supplements included inulin (*n* = 2) [43,44], oat flakes (*n* = 1) [40], isomalto-oligosaccharides (*n* = 1) [42], partially hydrolyzed guar gum (PHGG, *n* = 1) [45], fructo-oligosaccharides (FOS, *n* = 1) [41] and bran or mixtures of bran (*n* = 1) [39]. The characteristics of the included studies were documented in detail in Table 2.

### 3.3. Risk of Bias

**Figure 2:**
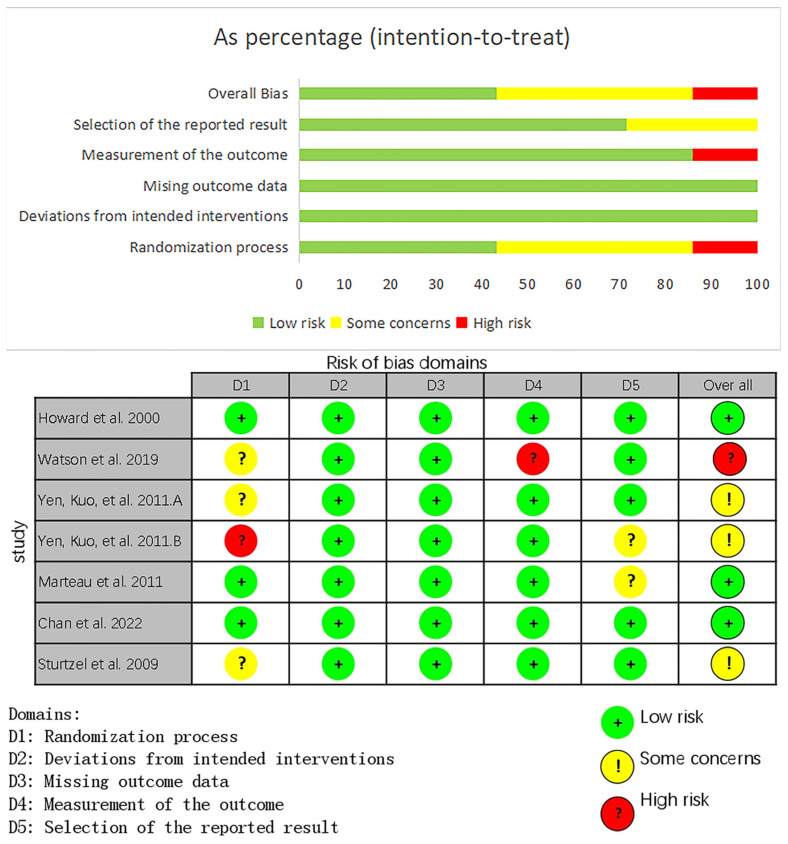
Summary plot of the risk-of-bias assessment for studies of constipation in the elderly [39,40,41,42,43,44,45].

A summary of the assessment of the risk of bias in studies of constipation is shown in Figure 2. The RoB 2.0 tool was employed to assess the risk of bias in the included studies. The original data and evaluation results of seven related studies were collected. Among the seven studies, one study was rated as high risk, three studies were moderate risk, and three studies were low risk. According to the comprehensive evaluation results, the overall risk level of this meta-analysis is medium.

### 3.4. Outcomes

#### 3.4.1. Stool Frequency

**Figure 3:**
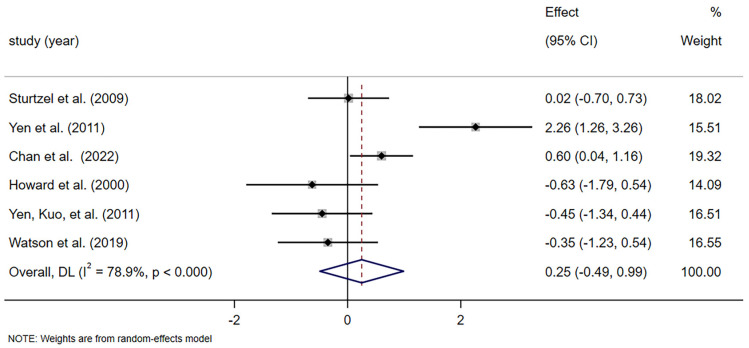
Meta-analysis of selected studies comparing dietary fiber and control for stool frequency [39,40,41,42,44,45].

Six studies with 137 patients reported continuous data on stool frequency [39,40,41,42,44,45]. Meta-analysis revealed no statistically significant effect of dietary fiber on stool frequency compared with control (SMD = 0.25, 95% CI [−0.488, 0.988], *p* = 0.507), with substantial heterogeneity (*I*^2^ = 78.9%, *p* = 0.000; Figure 3). Results of subgroup analyses similarly failed to detect a substantial elevation in weekly bowel movement frequency among subjects receiving prebiotic (SMD = 0.499, 95% CI [−0.547, 1.546], *p* = 0.350; *I*^2^ = 85%, *p* = 0.000; Figure S1.1) or non-prebiotic (SMD = −0.159, 95% CI [−0.769, 0.450], *p* = 0.609; *I*^2^ = 0.0%, *p* = 0.356; Figure S1.1) groups. Meanwhile, subgroup analyses suggested that the duration of treatment and participant numbers did not significantly affect stool frequency (Figures S1.2 and S1.3). Neither higher doses (≥10 g/d) (SMD = 0.473, 95% CI [−1.192, 2.139], *p* = 0.578; *I*^2^ = 89.8%, *p* = 0.000; Figure S1.4) nor low dietary fiber doses (<10 g/d) (SMD = 0.354, 95% CI [−0.211, 0.918], *p* = 0.219; *I*^2^ = 37.1%, *p* = 0.207; Figure S1.4) significantly affected stool frequency. In particular, we conducted sensitivity analyses on studies involving stool frequency as an intervention result, with the results remaining stable (Figure S3).

#### 3.4.2. Stool Dry Weight

**Figure 4:**
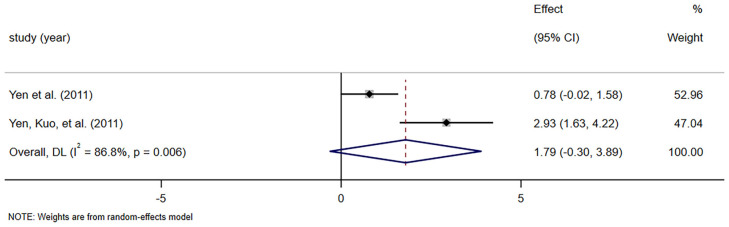
Meta-analysis of selected studies comparing dietary fiber and control for stool dry weight [41,42].

Two RCTs [41,42] involving 23 patients reported the effect of dietary fiber on stool dry weight in elderly constipation patients. No significant difference was found between the dietary fiber and control groups (SMD = 1.792, 95% CI [−0.303, 3.887], *p* = 0.094), with considerable heterogeneity (*I*^2^ = 86.8%, *p* = 0.006; Figure 4). Due to the limited number of trials available, sensitivity analyses and subgroup analyses were not performed.

#### 3.4.3. Frequency of Laxative or Enema Use

**Figure 5:**
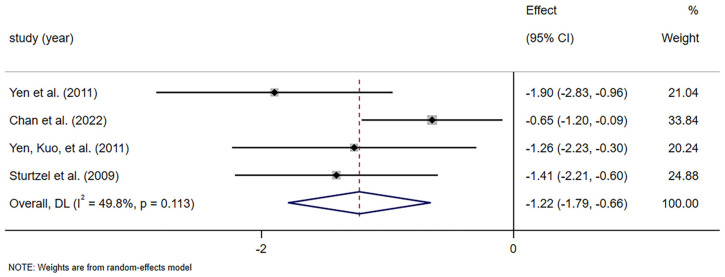
Meta-analysis of selected studies comparing dietary fiber and control for frequency of laxative or enema use [40,41,42,45].

Frequency of laxative or enema use was evaluated by four studies including 105 patients [40,41,42,45]. The results of the meta-analysis showed that dietary fiber supplementation significantly reduced the frequency of laxative or enema use in the elderly with constipation, with moderate heterogeneity (SMD = −1.224, 95% CI [−1.786, −0.662], *p* = 0.000; *I*^2^ = 49.8%, *p* = 0.113; Figure 5). In the subgroup analysis of dosage, it was observed that a dietary fiber supplementation of ≥10 g/d significantly reduced the frequency of laxative or enema use, with low heterogeneity (SMD = −1.592, 95% CI [−2.265, −0.918], *p* = 0.000; *I*^2^ = 0.00%, *p* = 0.358; Figure S2). A study indicated that participants in the dietary fiber supplementation group exhibited a significantly reduced usage of bowel medication compared to the control group [39]. No significant funnel plot asymmetry was detected (Figure S4).

#### 3.4.4. Intestinal Bifidobacteria Concentration

**Figure 6:**
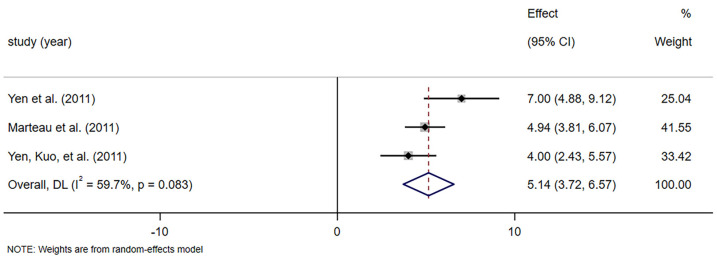
Meta-analysis of selected studies comparing dietary fiber and control for bifidobacteria concentration [41,42,43].

Three studies involving 73 participants assessed intestinal bifidobacteria levels [41,42,43]. Meta-analysis showed significantly higher numbers of intestinal bifidobacteria in the fiber supplement group than in the control group (SMD = 5.142, 95% CI [3.716, 6.568], *p* = 0.000; *I*^2^ = 59.7%, *p* = 0.083; Figure 6). Sensitivity analysis of intestinal bifidobacteria concentration demonstrated that the overall effect did not change, and the funnel plots indicated that there was no publication bias (Figure S5).

#### 3.4.5. Adverse Events

**Figure 7:**
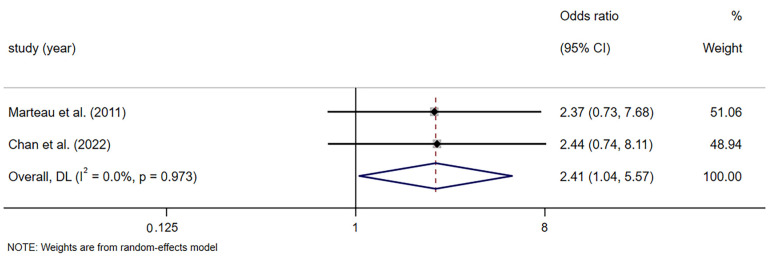
Meta-analysis of selected studies comparing fibers and controls for adverse events [43,45].

Two RCTs reported 36 adverse events: 23 in the fiber group and 13 in the placebo group, with most involving flatulence, bloating, or abdominal discomfort [43,45]. Combining the adverse reactions from the two trials, the meta-analysis findings revealed that the incidence of bowel symptoms was significantly higher with fiber supplementation compared to placebo (OR = 2.408, 95% CI [1.041, 5.571], *p* = 0.04; *I*^2^ = 0.0%, *p* = 0.973; Figure 7). Additionally, among the remaining three studies, there were no instances of severe adverse reactions associated with dietary fiber supplementation treatment. Notably, two studies did not provide any documentation of adverse events.

## 4. Discussion

This systematic review and meta-analysis synthesized the existing published evidence regarding the efficacy of fiber supplementation in managing chronic constipation among the elderly. The analysis included seven randomized controlled trials with a total of 187 participants from diverse geographic regions. The key findings suggest that while dietary fiber supplementation does not significantly improve stool frequency among the elderly, it substantially reduces laxative or enema use and enhances gut microbiota composition, particularly increasing bifidobacteria counts.

Prior investigations have examined the efficacy of fiber in alleviating constipation across diverse age demographics, spanning from children to adults. A recent systematic review and meta-analysis concluded that fiber supplementation is effective in managing constipation in adults [46]. Specifically, the study found that psyllium supplements and a dietary fiber dose of over 10 g/d were most effective in improving treatment compliance, stool consistency, and constipation, while a treatment duration of 4 weeks or more was found to be optimal for improvement in stool frequency and total gut transit time. The study conducted by Christodoulides et al. indicated that dietary fiber exerts a moderate effect on constipation, though efficacy and side effects may vary by dosage [47]. However, a meta-analysis by Piccoli et al. including nine randomized controlled trials (RCTs) demonstrated no statistically significant effects on bowel movement frequency, stool consistency, or treatment success. There is no scientific evidence that supplementing the diet of children with constipation with fiber is one treatment for the condition [48]. Collectively, these findings suggest age-specific responses to fiber and highlight the need for targeted research in older adults. Our findings suggest that dietary fiber may serve as an effective intervention in the management of constipation in older people.

Stool frequency is a diagnostic criterion for constipation; its improvement alone may not fully capture the therapeutic benefit of fiber [49]. However, there is a lack of a statistically significant improvement in stool frequency as shown in Figure 3. The same result was found in another systematic review [48]. Among the studies included, Yen et al. reported that the beneficial effects of isomalto-oligosaccharides on defecation frequency were shown with 8 wk supplementation in chronically constipated elderly people [42]. In contrast, Chan et al. found only a small, non-significant difference in stool frequency between the intervention and control groups [45]. Similarly, another study from Yen observed that fructo-oligosaccharide (FOS) supplementation had no effect on the frequency of spontaneous bowel movements [41]. We found some statistical heterogeneity in defecating frequency. Differences in the type of fiber may account for the heterogeneity of results in stool frequency reports [50]. However, the subgroup analysis revealed no significant difference in stool frequency between the prebiotic and non-prebiotic groups. Additionally, this heterogeneity could be influenced by the small sample sizes, dosage, and duration of the intervention diminishing the individual studies’ ability to detect effects, as well as discrepancies in outcome measurements, although no statistically significant difference was detected in the subgroup analysis. Another possible explanation is that the elderly participants included in this study primarily came from long-term care facilities and had potential issues with constipation, requiring the use of laxatives at baseline. In our study, we observed a reduction in the use of laxatives among participants in the dietary fiber group. This suggests that dietary fiber effectively alleviates tendencies towards constipation by reducing dependence on laxatives while maintaining similar bowel movement frequency and stool characteristics [51]. Our study also found that the effect of dietary fiber on stool dry weight was not statistically significant. This might be due to the limited number of trials (*n* = 2) and small sample size (*n* = 23). Laxatives are the most commonly used drugs for constipation because of their effectiveness and availability [52,53]. Studies have shown that approximately 50% of nursing home residents suffer from constipation, and 70% of them require laxatives [54,55]. The four studies included in this analysis demonstrated a significantly lower frequency of laxative use in the dietary fiber supplementation group compared to the control group. This finding supports previous literature suggesting that dietary fiber may reduce pharmacological dependency in older adults [56,57]. This outcome is clinically meaningful, particularly for older adults who are prone to polypharmacy, dependency, and medication-related side effects. The observed reduction in pharmacological reliance supports the role of dietary fiber as a non-pharmacologic adjunct therapy and highlights its potential to reduce healthcare costs and improve medication safety in geriatric care [58].

Another key finding was the significant increase in fecal bifidobacteria following dietary fiber supplementation as presented in Figure 6. This supports the prebiotic role of certain dietary fiber, particularly inulin and FOS, which are known to selectively stimulate the growth of beneficial gut microbiota [59,60]. Bifidobacteria play a critical role in maintaining gut barrier integrity, modulating inflammation, and regulating bowel function [61]. Decreased concentrations of fecal bifidobacteria in the elderly, which may contribute in part to intestinal dysfunction and thus constipation [62]. This result suggests that dietary fiber may improve constipation in older adults by altering the microbial composition of the gut. However, more research is needed to delve deeper into the effect of dietary fiber on gut microbial composition and to further understand how this effect correlates with improvement in constipation symptoms.

However, the benefits of dietary fiber must be weighed against its tolerability. Our results indicated a higher incidence of gastrointestinal side effects, such as bloating and flatulence, in the dietary fiber group. These effects were more prominent in studies involving inulin and PHGG, both of which are highly fermentable fibers known to produce gas during bacterial fermentation, resulting in bloating [63]. These findings are consistent with previous reports highlighting that rapid increases in fiber intake without adequate hydration or gradual adaptation may lead to discomfort [64,65]. Future interventions should consider patient-specific tolerance and incorporate gradual dose escalation protocols.

Strengths of this review include its strict adherence to the PRISMA guidelines, covering multiple electronic databases with no language restrictions. This rigorous approach ensured that a wide range of relevant studies were captured, reducing the risk of publication bias. Furthermore, the authors used a standardized template for data extraction and assessed risk of bias using the RoB 2.0 tool, a systematic approach that enhanced the reliability of the extracted data. However, this review also has certain limitations. The total number of included studies was relatively small, which may reduce the generalizability of the findings. We also acknowledged that not all studies contributed to every outcome analysis due to reporting limitations. In addition, there was substantial heterogeneity in the types of dietary fiber used, their dosages, administration forms, and durations of intervention, making direct comparisons and pooled effect estimation challenging [66]. The variability in study designs and intervention protocols introduces the potential for confounding factors that may influence the observed effects of dietary fiber supplementation on chronic constipation outcomes. Factors such as baseline dietary habits, concomitant medications, and comorbidities could affect the results but may not have been adequately controlled for across studies [67]. Moreover, possibility of publication bias despite a comprehensive literature search. Statistically significant results are more likely to be published, leading to an overestimation of the outcome of dietary fiber supplementation on chronic constipation.

The wide variety of types of dietary fiber poses an additional challenge to the research effort. Previous evidence indicated that the efficacy of dietary fiber is modulated by both dosage and duration of intervention [46]. In our subgroup analysis of dosage, daily intakes ≥ 10 g were associated with a significant reduction in laxative/enema use, whereas lower doses did not reach statistical significance. Conversely, stool frequency did not improve even at higher doses. Meanwhile, subgroup analyses showed that the duration of treatment had no significant effect on stool frequency. One plausible explanation is that stool frequency may be influenced by individual factors in elderly people, such as the use of laxatives [68]. To enhance the guidance of clinical application and the development of treatment strategies, future studies should prioritize investigating the impact of fiber type, dosage, and treatment duration on potential benefits. Further exploration into the use of fiber for managing constipation in older adults is warranted to deepen our understanding of its safety and efficacy, as well as to offer improved treatment options for this population.

## 5. Conclusions

This systematic review and meta-analysis provided updated evidence on the role of dietary fiber supplementation in managing chronic constipation among the elderly. While the overall effect on stool frequency was not statistically significant, dietary fiber intake was associated with a marked reduction in laxative or enema use, and a significant increase in intestinal bifidobacteria concentrations, suggesting a beneficial impact on gut microbiota and dependency on pharmacological interventions. However, the findings should be interpreted with caution due to heterogeneity, limited sample sizes, and variability in fiber types and dosages across the included trials. Therefore, to further investigate the role of fiber supplementation in the management of constipation, large-scale, meticulously designed, parallel-group, large RCTs comparing fiber with known conventional and effective therapies are needed. These studies should also explore the dose-response relationship, duration of supplementation, and the long-term safety profile of dietary fiber in elderly populations. Overall, this meta-analysis supports the use of dietary fiber as a safe and potentially effective non-pharmacological strategy for alleviating chronic constipation in older adults, with implications for reducing polypharmacy and improving quality of life.

## Figures and Tables

**Table 1:** PICOS criteria for inclusion of studies.

Parameter	Criteria
Participants (P)	Constipated patients over the age of 50 of any ethnicity and geographic area
Interventions (I)	Any type of dietary fiber supplement
Comparisons (C)	Placebo or habitual diet or any pharmacological or non-pharmacological interventions
Outcomes (O)	Stool frequency, stool consistency, stool weight, gastrointestinal symptoms, use of laxatives or enemas, and adverse events
Study design (S)	Randomized controlled trials (RCTs) in either parallel or crossover design

**Table 2:** Summary of the studies of dietary fiber supplementation on chronic constipation in the elderly.

First Author/ Year	Study Design	Number of Participants	Age (Range) (Years)	Diagnostic Criteria	Duration of Therapy	Intervention/Dose	Form	Outcomes
Sturtzel 2009 [40]	Double-blind, parallel	30	57–98	Not mentioned	Twelve weeks	Ordinary oat flakes vs habitual diet; 7–8 g oat-bran/d	Solid	The usage of laxatives; bodyweight; stool frequency
Yen 2011 [42]	Double-blind, crossover	13	82.5 ± 1.9	Not mentioned	Eight weeks	Isomalto-oligosaccharide vs. Placebo; 5 g/d for the first 7 d 22 g/d for the rest of time	Liquid	Fecal bacteria; bowel function; fecal short-chain fatty acids; biochemical indices
Marteau 2011 [43]	Double-blind, parallel	50	50–70	Rome definition	Four weeks	Native inulin vs. Placebo; 15 g/d	Sachet	Microbiological analyses; bowel symptoms; bowel motor function parameters
Chan 2022 [45]	Single-blind, parallel	52	83.9 ± 7.6	Rome III criteria	Four weeks	PHGG vs. Placebo; 5 g PHGG/d	Liquid	Frequency of laxative use; fecal consistency; fecal frequency; fecal
Howard, 2000 [39]	Double-blind, parallel	12	61–80	Two or fewer stools/week	Five months	Bran mixture vs. Placebo; ≥3 tablespoons/d	Liquid	Number of bowel movements; number of bowel medications
Watson 2019 [44]	Double-blind, crossover	20	62 (from 51 to 74)	Self-reported	Five weeks	Inulin vs. placebo; 10 g/day	Sachet	Stool frequency; consistency; fecal microbiota
Yen 2011 [41]	Double-blind, crossover	10	74.0 ± 3.3	Taking medication for constipation or ≤3 spontaneous bowel movements/week or straining with a bowel movement without medication	Four weeks	FOS vs. placebo; 5 g/d for the first 7 d 10 g/d for 8–28 days	Liquid	Fecal bifidobacteria; lipid peroxidation index; indices of nutritional status

Note: PHGG, partially hydrolyzed guar gum; FOS, fructo-oligosaccharides.

## Data Availability

The original contributions presented in the study are included in the article/Supplementary Materials. Further inquiries can be directed to the corresponding authors.

## Supplementary Materials

The following supporting information can be downloaded at: https://www.mdpi.com/article/10.3390/foods14132315/s1, Figure S1.1: Subgroup analysis of stool frequency (prebiotic or non-prebiotic); Figure S1.2: Subgroup analysis of stool frequency (sample size); Figure S1.3: Subgroup analysis of stool frequency (sample size); Figure S1.4: Subgroup analysis of stool frequency(dose); Figure S2: Subgroup analysis of frequency of laxative or enema use(dose); Figure S3: Sensitivity analysis and funnel plot of stool frequency; Figure S4: Sensitivity analysis and funnel plot of laxative or enema use; Figure S5: Sensitivity analysis and funnel plot of intestinal bifidobacteria concentration; Table S1: PRISMA_2020_checklist; Table S2: Detailed search strategies for each database; Table S3: Revised Cochrane risk-of-bias tool for randomized trials (ROB 2.0).
