# Supplementary material for: Effects of Dietary Fiber Supplementation on Chronic Constipation in the Elderly: A Systematic Review and Meta-Analysis of Randomized Controlled Trials
Source: Foods. 2025 Jun 30;14(13):2315. doi: 10.3390/foods14132315 (PMC12249261; doi:10.3390/foods14132315)
Supplement: Supplementary file 1 [file foods-14-02315-s001.zip › Supplementary Figure.pdf]

Supplementary Figure S1.

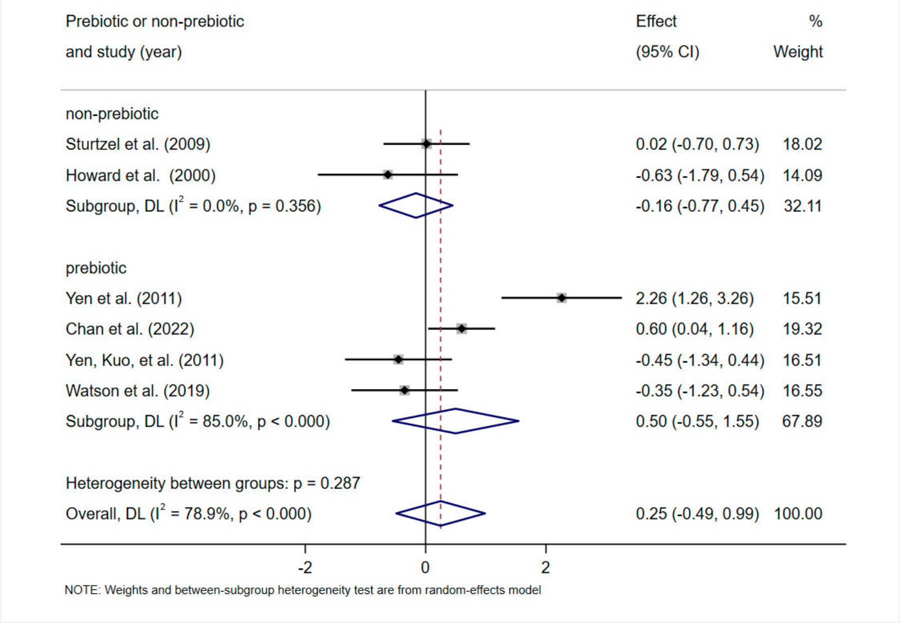

Figure S1.1 Subgroup analysis of stool frequency (prebiotic or non-prebiotic)

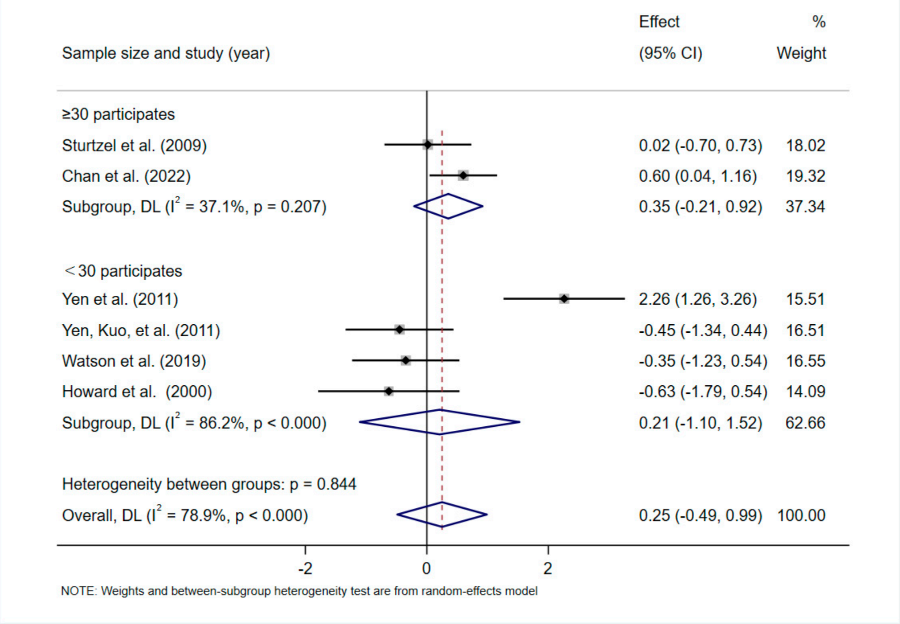

Figure S1.2 Subgroup analysis of stool frequency(sample size)

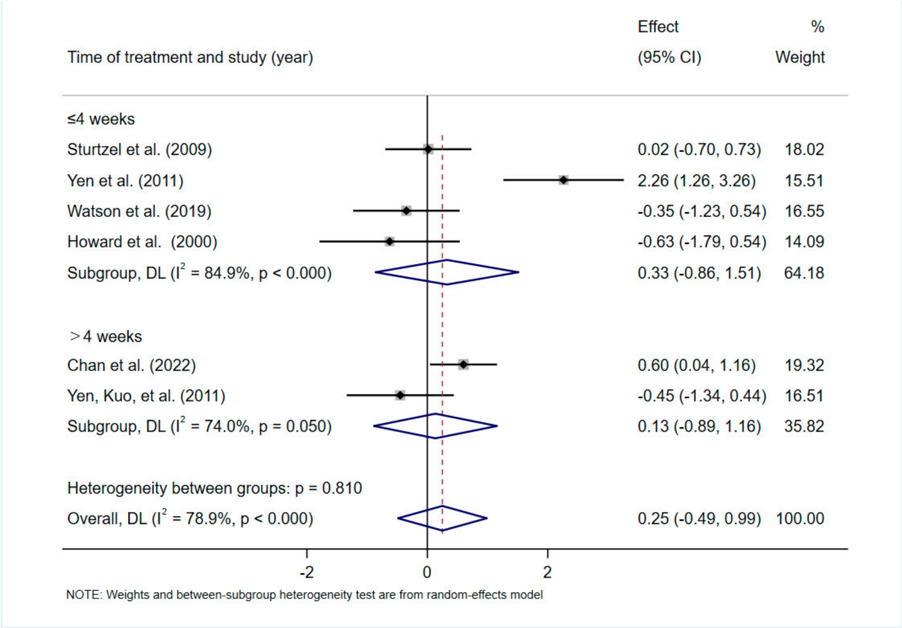

Figure S1.3 Subgroup analysis of stool frequency(time of treatment)

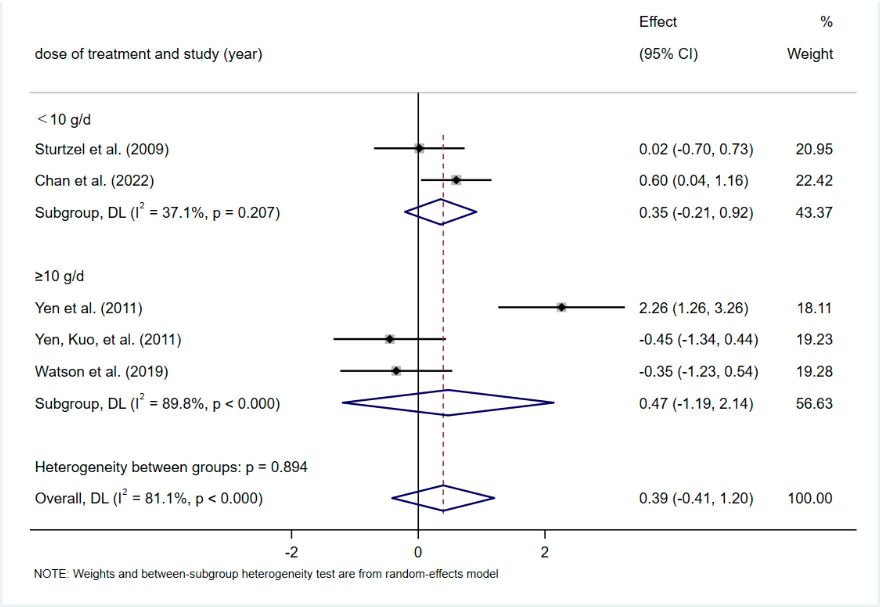

Figure S1.4 Subgroup analysis of stool frequency(dose)

Supplementary Figure S2.

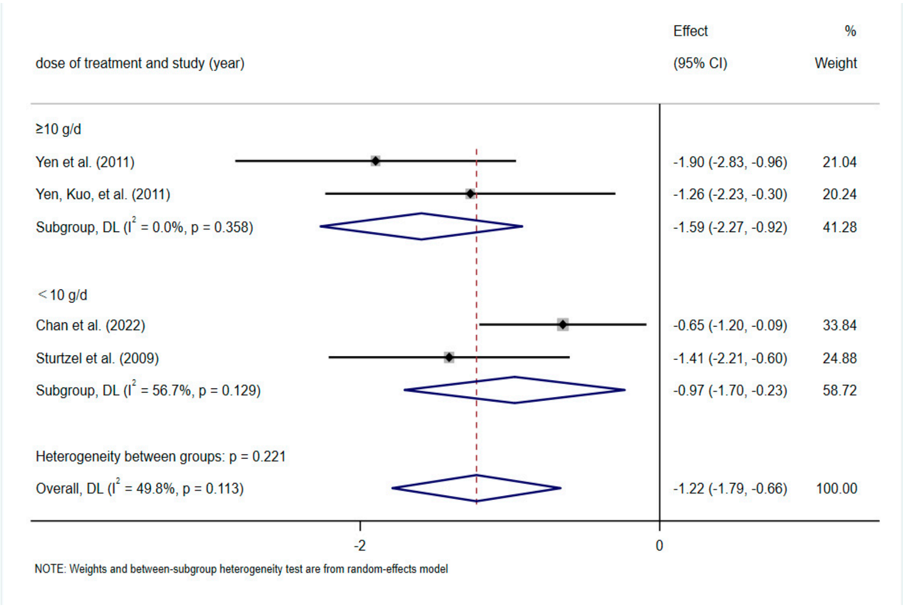

Figure S2 Subgroup analysis of frequency of laxative or enema use(dose)

Supplementary Figure S3.

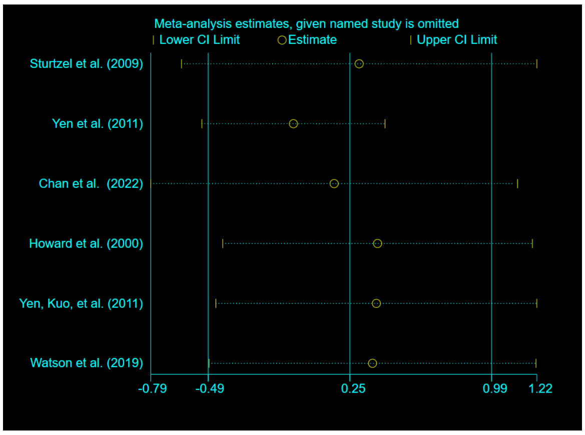

Figure S3.1 Sensitivity analysis of stool frequency

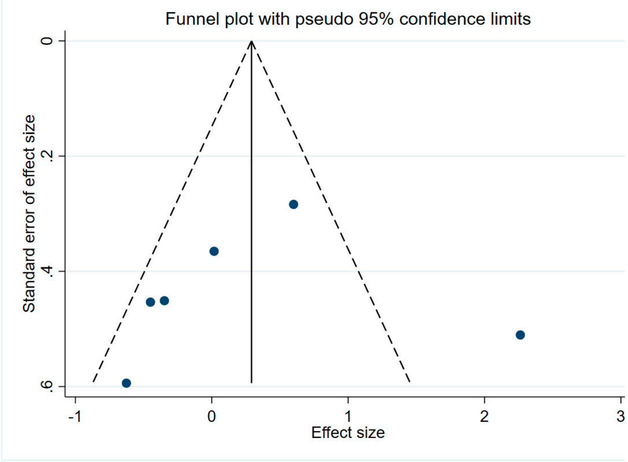

Figure S3.2 Funnel plot of stool frequency

Supplementary Figure S4.

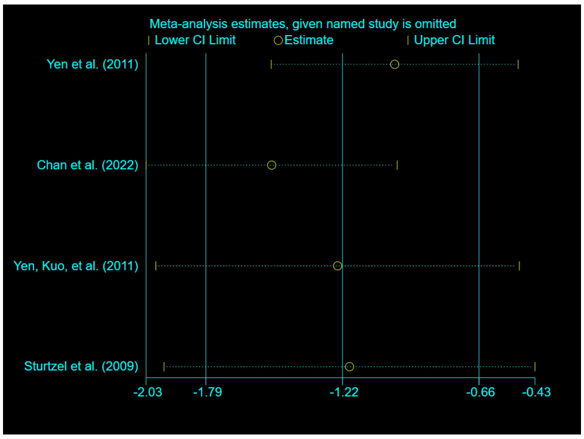

Figure S4.1 Sensitivity analysis of frequency of laxative or enema use

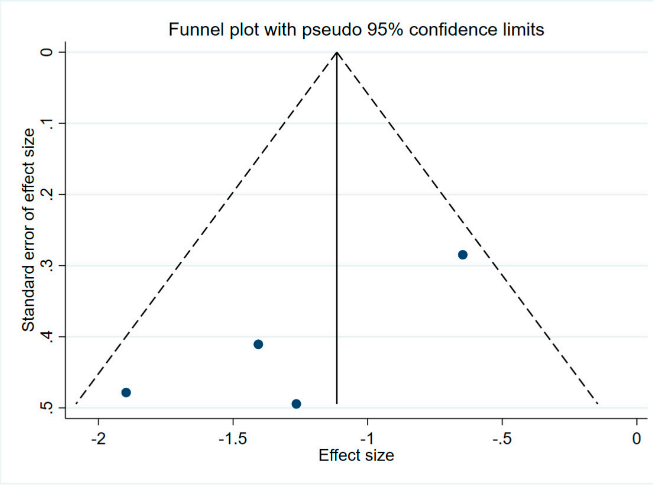

Figure S4.2 Funnel plot of frequency of laxative or enema use

Supplementary Figure S5.

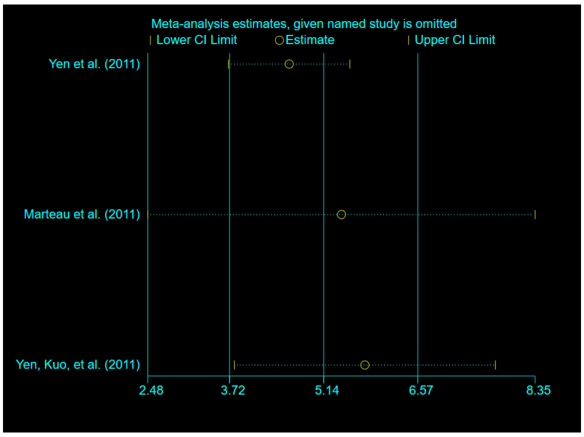

Figure S5.1 Sensitivity analysis of intestinal bifidobacteria concentration

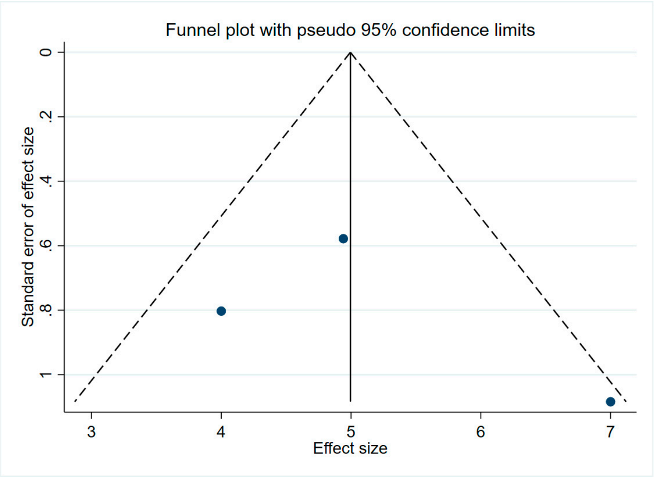

Figure S5.2 Funnel plot of intestinal bifidobacteria concentration
