# Supplementary material for: Effects of Dietary Fiber Supplementation on Chronic Constipation in the Elderly: A Systematic Review and Meta-Analysis of Randomized Controlled Trials
Source: Foods. 2025 Jun 30;14(13):2315. doi: 10.3390/foods14132315 (PMC12249261; doi:10.3390/foods14132315)
Supplement: Supplementary file 1 [file foods-14-02315-s001.zip › Supplementary Table S2 .pdf]

Supplemental Table S2

<table>
<tr><td>Pubmed=7</td><td colspan="2">((((Aged[Title/Abstract])OR(Elderly[Title/Abstract]))AND(((Constipation[Title/Abstract])OR(Dyschezia[Title/Abstract]))OR(Colonic Inertia[Title/Abstract])))AND((((((((((Dietary Fiber[Title/Abstract])OR(Dietary Fibers[Title/Abstract]))OR(Fibers,Dietary[Title/Abstract]))OR(Fiber,Dietary[Title/Abstract]))OR(Wheat Bran[Title/Abstract]))OR(Bran, Wheat[Title/Abstract]))OR(Brans, Wheat[Title/Abstract]))OR(Wheat Brans[Title/Abstract]))OR (Roughage[Title/Abstract]))OR(Roughages[Title/Abstract])))AND(randomized controlled trials)</td></tr>
<tr><td>Embase=18</td><td colspan="2">('constipation'/exp OR 'constipation' OR 'dyschezia':ab,ti OR 'colonic inertia':ab,ti) AND ('aged'/exp OR 'aged' OR elderly:ab,ti) AND ('dietary fiber'/exp OR 'dietary fiber' OR 'dietary fibers':ab,ti OR 'fibers, dietary':ab,ti OR 'fiber, dietary':ab,ti OR 'wheat bran':ab,ti OR 'bran, wheat':ab,ti OR 'brans, wheat':ab,ti OR 'wheat brans':ab,ti OR 'roughage':ab,ti OR 'glomerulonephritis, membranous':ab,ti OR 'roughages':ab,ti) AND ('randomized controlled trials ':ab,ti)</td></tr>
<tr><td>Scopus=129</td><td colspan="2">(TITLE-ABS-KEY (aged OR elderly) AND TITLE-ABS-KEY (constipation OR dyschezia) AND TITLE-ABS-KEY ("Dietary fiber" OR "dietary fibers" OR "fibers dietary" OR "fiber dietary" OR "wheat bran" OR "bran wheat" OR "brans wheat" OR "wheat brans" OR roughage OR roughages) AND TITLE-ABS-KEY ("Randomized controlled" AND trials OR "clinical trials randomized" OR "trials randomized clinical" OR "controlled clinical trials randomized"))</td></tr>
<tr><td rowspan="5">Web of science=105</td><td>#1</td><td>TS= (Aged OR Elderly*)</td></tr>
<tr><td>#2</td><td>TS= (Constipation OR Dyschezia* OR Colonic Inertia)</td></tr>
<tr><td>#3</td><td>TS= (Dietary Fiber OR Dietary Fibers* OR Fibers, Dietary OR Fiber, Dietary* OR Wheat Bran OR Bran, Wheat* OR Wheat Brans OR Roughage* OR Roughage OR Roughages*)</td></tr>
<tr><td>#4</td><td>TS= (Randomized Controlled Trials)</td></tr>
<tr><td>#5</td><td>#1AND#2AND#3AND#4</td></tr>
<tr><td rowspan="5">The Cochrane Library=74</td><td>#1</td><td>MeSH descriptor: [Aged] in all MeSH products</td></tr>
<tr><td>#2</td><td>(Elderly): ti,ab,kw</td></tr>
<tr><td>#3</td><td>#1 or #2</td></tr>
<tr><td>#4</td><td>MeSH descriptor: [Constipation] explode all trees</td></tr>
<tr><td>#5</td><td>(Dyschezia): ti,ab,kw OR (Colonic Inertia): ti,ab,kw</td></tr>
</table>

	#6	#4 or #5
	#7	MeSH descriptor: [Dietary Fiber] explode all trees
	#8	(Dietary Fibers): ti,ab,kw OR (Fibers, Dietary): ti,ab,kw OR (Fiber, Dietary): ti,ab,kw OR (Wheat Bran): ti,ab,kw OR (Bran, Wheat): ti,ab,kw
	#9	(Brans, Wheat): ti,ab,kw OR (Wheat Brans): ti,ab,kw OR (Roughage): ti,ab,kw OR (Roughages): ti,ab,kw
	#10	#7 or #8 or #9
	#11	#3 and #6 and #10
