# Supplementary material for: Effects of Dietary Fiber Supplementation on Chronic Constipation in the Elderly: A Systematic Review and Meta-Analysis of Randomized Controlled Trials
Source: Foods. 2025 Jun 30;14(13):2315. doi: 10.3390/foods14132315 (PMC12249261; doi:10.3390/foods14132315)
Supplement: Supplementary file 1 [file foods-14-02315-s001.zip › Supplementary Table S3.pdf]

Supplemental Table S3

Revised Cochrane risk-of-bias tool for randomized trials (ROB 2.0)

**Domain**	**Signalling question**	**Response**
Domain 1: Bias arising from the randomization process	1.1 Was the allocation sequence random?	Y/PY/PN/N/NI
	1.2 Was the allocation sequence concealed until participants were enrolled and assigned to interventions?	Y/PY/PN/N/NI
	1.3 Did baseline differences between intervention groups suggest a problem with the randomization process?	Y/PY/PN/N/NI
	Risk of bias judgement	Low/High/Some concerns
Domain 2: Bias due to deviations from intended interventions	2.1. Were participants aware of their assigned intervention during the trial?	Y/PY/PN/N/NI
	2.2. Were carers and people delivering the interventions aware of participants assigned intervention during the trial?	Y/PY/PN/N/NI
	2.3. If Y/PY/NI to 2.1 or 2.2: Were there deviations from the intended intervention that arose because of the experimental context?	NA/Y/PY/PN/N/NI
	2.4 If Y/PY to 2.3: Were these deviations likely to have affected the outcome?	NA/Y/PY/PN/N/NI
	2.5. If Y/PY/NI to 2.4: Were these deviations from intended intervention balanced between groups?	NA/Y/PY/PN/N/NI
	2.6 Was an appropriate analysis used to estimate the effect of assignment to intervention?	Y/PY/PN/N/NI
	2.7 If N/PN/NI to 2.6: Was there potential for a substantial impact (on the result) of the failure to analyze participants in the group to which they were randomized?	NA/Y/PY/PN/N/NI
	Risk of bias judgement	Low/High/Some concerns
Domain 3: Bias due to missing outcome data	3.1 Were data for this outcome available for all, or nearly all, participants randomized?	Y/PY/PN/N/NI
	3.2 If N/PN/NI to 3.1: Is there evidence that result was not biased by missing outcome data?	NA/Y/PY/PN/N/NI
	3.3 If N/PN to 3.2: Could missingness in the outcome depend on its true value?	NA/Y/PY/PN/N/NI
	3.4 If Y/PY/NI to 3.3: Is it likely that missingness in the outcome depended on its true value?	NA/Y/PY/PN/N/NI
	Risk of bias judgement	Low/High/Some concerns
Domain 4:	4.1 Was the method of measuring the outcome inappropriate?	Y/PY/PN/N/NI

Bias in measurement of the outcome	4.2 Could measurement or ascertainment of the outcome have differed between intervention groups?	Y/PY/PN/N/NI
	4.3 Were outcome assessors aware of the intervention received by study participants?	Y/PY/PN/N/NI
	4.4 If Y/PY/NI to 4.3: Could assessment of the outcome have been influenced by knowledge of intervention received?	NA/Y/PY/PN/N/NI
	4.5 If Y/PY/NI to 4.4: Is it likely that assessment of the outcome was influenced by knowledge of intervention received?	NA/Y/PY/PN/N/NI
	Risk of bias judgement	Low/High/Some concerns
Domain 5: Bias in selection of the reported result	5.1 Were the data that produced this result analyzed in accordance with a pre-specified analysis plan that was finalized before unblinded outcome data were available for analysis?	Y/PY/PN/N/NI
	5.2 ... multiple eligible outcome measurements (e.g. scales, definitions, time points) within the outcome domain?	Y/PY/PN/N/NI
	5.3 ... multiple eligible analyses of the data?	Y/PY/PN/N/NI
	Risk of bias judgement	Low/High/Some concerns
Overall bias	Risk of bias judgement	Low/High/Some concerns
